# Permanent Sensorineural Deafness in a Patient with Chronic Myelogenous Leukemia Secondary to Intracranial Hemorrhage

**DOI:** 10.1155/2013/894141

**Published:** 2013-12-03

**Authors:** Sakshi Kapur, Michael Wax, Levin Miles, Adnan Hussain

**Affiliations:** ^1^Department of Internal Medicine, Overlook Medical Center, 99 Beauvoir Avenue, Summit, NJ 07902, USA; ^2^Division of Hematology and Oncology, Overlook Medical Center, Summit, NJ 07902, USA; ^3^Division of Pathology, Overlook Medical Center, Summit, NJ 07902, USA

## Abstract

A 52-year-old male presented with tinnitus and fullness in left ear for one day. Workup revealed a white blood cell count of 685 × 10^3^/*μ*L with marked increase in granulocyte series and myeloid precursors on peripheral smear. The initial impression was chronic myelogenous leukemia with hyperleukocytosis, and patient was started on hydration, hydroxyurea, and allopurinol. Patient tolerated bone marrow biopsy well but continued to bleed excessively from the biopsy site. Results confirmed Philadelphia chromosome positive chronic myelogenous leukemia (chronic phase). On day three of hospitalization, patient developed sudden slurred speech along with shaking movements involving extremities. Magnetic resonance imaging revealed multiple hemorrhages throughout the brain. Hydroxyurea was continued until insurance coverage for nilotinib was getting approved. On day nine of hospitalization, patient developed sudden bilateral sensorineural deafness. Repeat magnetic resonance imaging revealed multiple new hemorrhages throughout the brain. Computer tomography of the temporal bones showed inflammatory changes in right and left mastoid cells. Nilotinib was started on day eleven of hospitalization. Patient's white blood cell count continued to decrease, but there was no improvement in hearing. Four months later, patient was treated with bilateral transmastoid cochlear implants. This case highlights permanent deafness as a hemorrhagic complication secondary to chronic myelogenous leukemia.

## 1. Introduction

Sudden hearing loss as an initial manifestation of an underlying hematological disease is rare. The pathogenesis of this clinical symptom appears to be complex. We report a case of permanent bilateral sensorineural deafness in a patient with newly diagnosed chronic myelogenous leukemia (chronic phase) secondary to intracranial bleeding.

## 2. Case Report 

A 52-year-old Caucasian male presented with tinnitus and fullness in left ear for one day. He complained of dizziness but denied any hearing loss or earache. He reported a 10 pound weight loss over the last three months. He also complained of occasional night sweats and progressively worsening lethargy for six months prior to hospitalization.

Physical examination revealed an average sized male with no acute distress. Vital signs were within normal limits. There was no lymph node enlargement in neck, supraclavicular fossa, axillary, inguinal, and femoral regions. Both liver and spleen were enlarged on percussion, measuring 14.8 cm and 8.0 cm, respectively. Cranial nerves 2–12 were intact and motor strength was equal in all four extremities. Bilateral external auditory canal and tympanic membrane were assessed to be normal. No nystagmus was detected.

**Figure 1:**
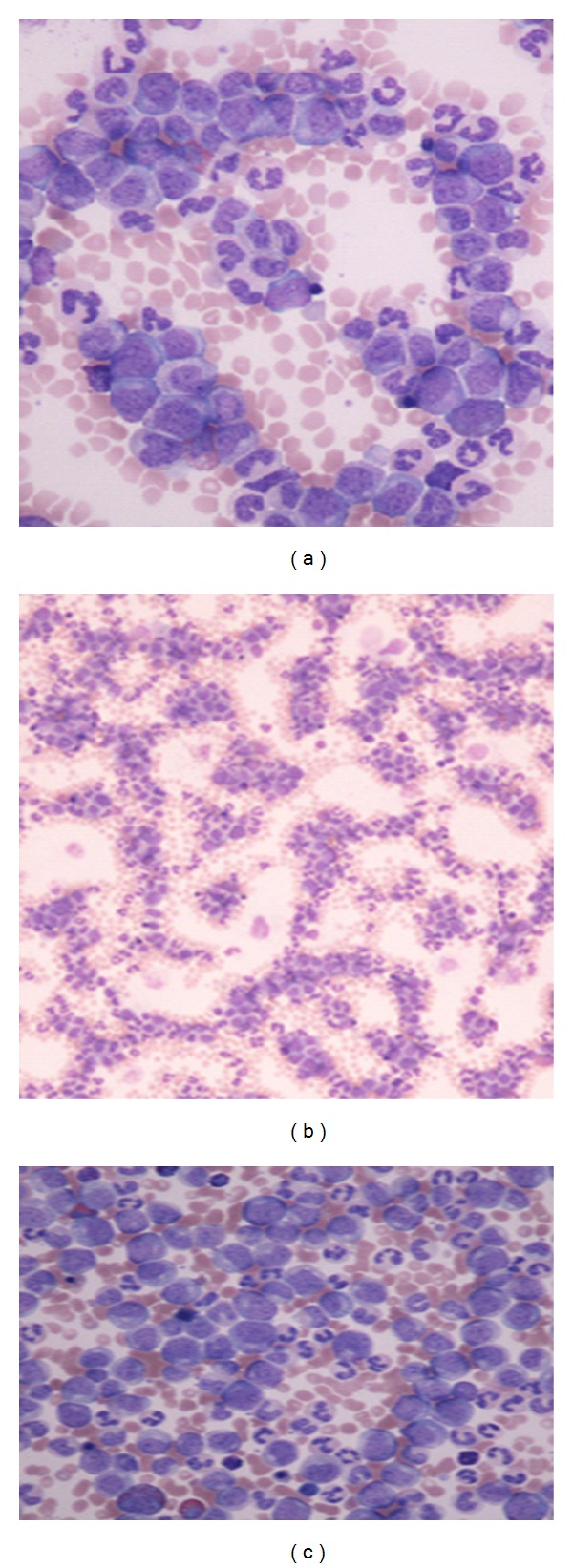
Peripheral smear showing marked increase in granulocyte and myeloid precursors with promyelocytes, myelocytes, metamyelocytes, and blast cells.

Workup revealed a hemoglobin count of 9.1 g/dL, white blood cell count of 685 × 10^3^/*μ*L, and a platelet count of 164 × 10^3^/*μ*L. Peripheral smear showed a marked increase in the granulocyte series and myeloid precursors with some blast cells, promyelocytes, myelocytes, and metamyelocytes (Figure 1).

Further workup revealed an elevated lactate dehydrogenase level of 1025 U/L (suggestive of excessive myeloid proliferation), uric acid level of 9.8 mg/dL, and leukocyte alkaline phosphatase level of zero.

The initial impression was chronic myelogenous leukemia (CML) with hyperleukocytosis. A bone marrow aspiration and biopsy were performed. Although the patient tolerated biopsy well, he continued to bleed excessively from the biopsy site. He was started on hydration, hydroxyurea (1 gm thrice a day), and allopurinol (300 mg twice a day). His urine was alkalinized with intravenous bicarbonate. On day two of hospitalization, the patient's white blood cell count dropped to 646 × 10^3^/*μ*L, and his uric acid was down to 5.8 mg/dL.

**Figure 2:**
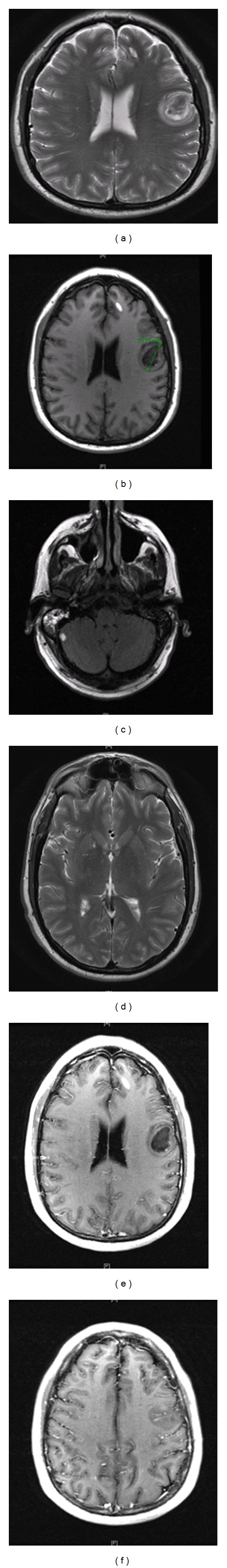
Magnetic resonance imaging of head showing multiple foci of abnormal signal compatible with blood products of different maturity, the dominant focus is measuring 3.1 cm in left frontal lobe, presumably a focus of most acute hemorrhage, and posterior fossa contains a single focus of abnormal T1 signal presumably of the same etiology.

Next day, patient developed sudden slurred speech along with shaking movements involving all extremities. Cranial magnetic resonance imaging revealed multiple hemorrhages throughout the brain, with most prominent hemorrhage in left peri-insular region (Figure 2). Cranial magnetic resonance angiogram showed no significant stenosis or aneurysm. Since the white blood cell count was still very elevated (525 × 10^3^/*μ*L), we decided to increase the dose of hydroxyurea (2 gm four times a day). Repeat blood work showed an international normalized ratio of 1.60 (prothrombin time (PT): 19.5 sec and activated PTT 34.2 sec).

**Figure 3:**
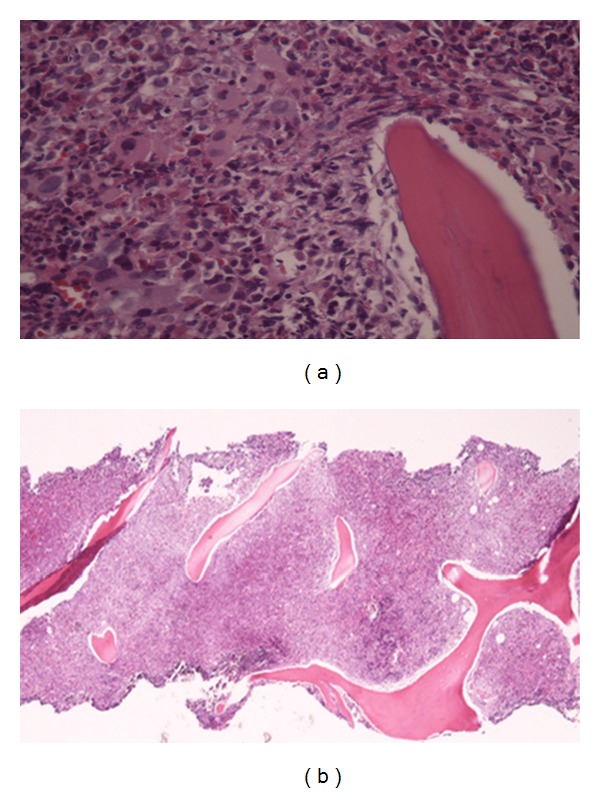
Bone marrow biopsy showing a hyper cellular marrow with increased myeloid cells in all maturation stages.

Bone marrow biopsy results revealed a hyper cellular marrow (>95% cellularity), increased myeloid cells in all maturation stages, M : E ratio of >10 : 1, and blasts of 9% (Figure 3). The fluorescence in situ hybridization (FISH) technique was positive for BCR-ABL fusion gene. This confirmed the diagnosis of CML (chronic phase), and we decided to start the patient on nilotinib. Hydroxyurea was continued until insurance coverage for nilotinib was approved.

**Figure 4:**
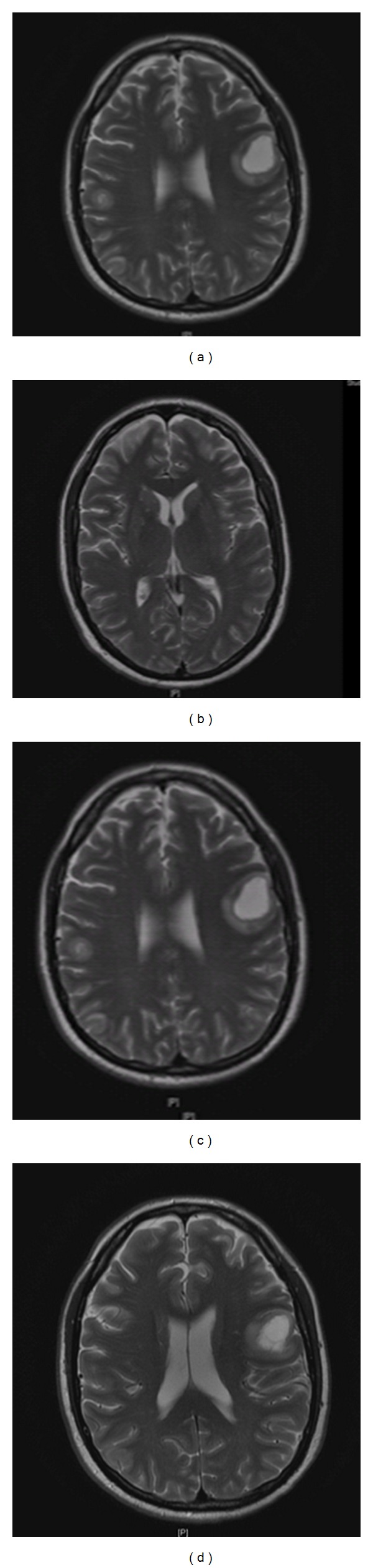
Magnetic resonance imaging of head showing multiple new acute hemorrhages in gray-white junction, mainly seen in the intra- and supratentorial space, measuring 2 cm or less.

**Figure 5:**
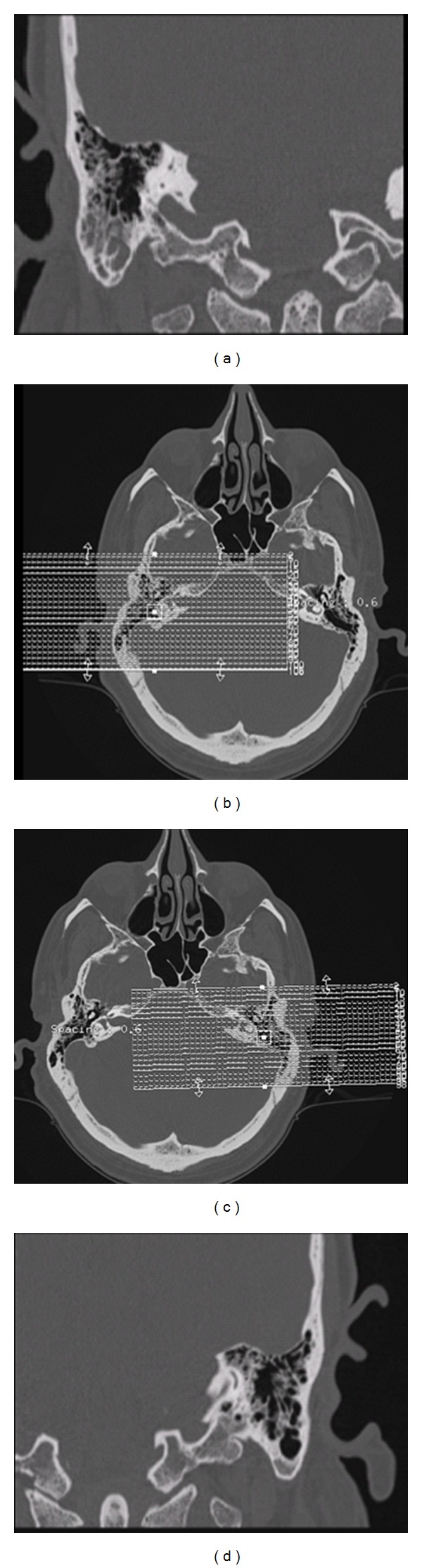
Computer tomography of temporal bones showing marked inflammatory changes in right mastoid cells ((a), (b)) and mild inflammatory changes seen in left mastoid cells ((c), (d)).

On day nine of hospitalization, patient developed sudden bilateral hearing loss. Physical examination was compatible with sensorineural deafness. Patient's white blood cell count had dropped to 62.9 × 10^3^/*μ*L and platelet count was 133 × 10^3^/*μ*L. Repeat magnetic resonance imaging revealed multiple new hemorrhages throughout the brain (Figure 4). Computer tomography of the temporal bones showed marked inflammatory changes in right mastoid cells and mild inflammatory changes in left mastoid cells. However, bilateral auditory canal, semicircular canals, middle ear cavity, and cochlea appeared to be normal (Figure 5). Repeat international normalized ratio was 1.39 (PT: 21 sec and activated PTT: 32.4 sec). Patient also developed a rash on his face and arms which was thought to be secondary to allopurinol. Since patient's white blood cell count had dropped to 62.9 × 10^3^/*μ*L, we decided to stop the allopurinol.

Patient's white blood cell count continued to decrease. Hepatosplenomegaly was resolving but there was no improvement in hearing. Nilotinib was started on day eleven of hospitalization. On the following day, patient's white blood cell count dropped to 4170/*μ*L, so we decided to hold the hydroxyurea. On day fourteen of hospitalization patent's white blood cell count further dropped to 890/*μ*L. Nilotinib was held and patient was started on filgrastim.

**Figure 6:**
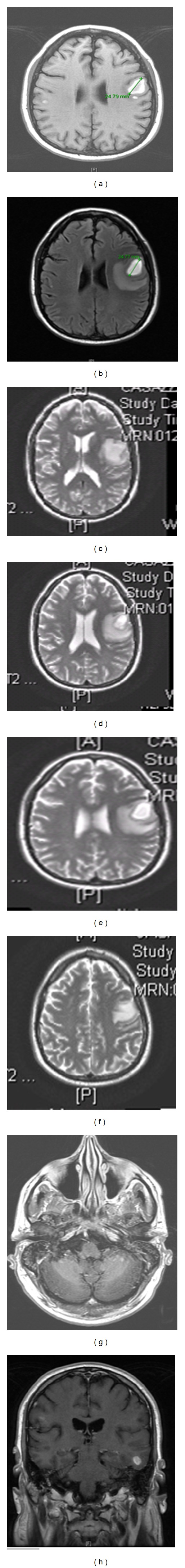
Magnetic resonance imaging (on discharge) showing improvement in multiple foci of hemorrhage, and dominant focus within the left frontal lobe subcortical matter appears to be stable with adjacent edema.

Although, the blood counts started improving, there was no improvement in hearing (Figure 6). Patient was discharged from the hospital and was asked to follow up, in order to restart nilotinib once his counts stabilized. Four months later, the patient continued to have bilateral sensorineural deafness and was treated with bilateral transmastoid cochlear implants. Six months later, a repeat bone marrow biopsy showed no evidence of residual CML

## 3. Discussion

Deafness as an initial manifestation of CML is rare. Very few cases have been reported in literature [1–4]. The pathogenesis of this clinical symptom appears to be complex and includes various mechanisms such as leukostasis, leukemic infiltration of cochlea, hyperviscosity syndrome, thrombohemorrhagic complications, and infections.

Hyperleukocytosis is defined as total leukemic cell count of greater than 100 × 10^3^/*μ*L. In contrast, leukostasis is a medical emergency, characterized by an extremely elevated blast cell count and symptoms of decreased tissue perfusion. Early treatment is warranted as the untreated mortality can be as high as 40 percent. In general, symptoms of leukostasis are more common in leukemia's with large, poorly deformable blasts, such as acute myeloid leukemia. In chronic lymphocytic leukemia a significant proportion of patients present with hyperleukocytosis. Symptoms of leukostasis are rare unless the WBC count exceeds 400 × 10^3^/*μ*L. In CML, the median WBC count is approximately 100 × 10^3^/*μ*L. These mainly include the segmented neutrophils, metamyelocytes, and myelocytes. Symptoms of leukostasis are very rare in chronic phase of CML but can occasionally occur in blast crisis with very elevated blast counts. Complications include visual changes, headache, gait instability, coma, priapism, and bowel infarction. Although few, cases of deafness in patients with CML secondary to hyperleukocytosis/leukostasis have been reported [2–4]. Early cytoreduction with leukopheresis and/or chemotherapy led to reversal of symptoms in some patients [5–7]. However, small leukocytic thrombi secondary to hyperleukocytosis can cause occlusion of blood vessels and their small branches causing infarction and permanent end organ damage. Endothelial damage secondary to leukemic cells has also been suggested in the pathogenesis of leukocytic thrombi [8, 9]. Our patient developed sudden deafness when the white blood cell count had dropped to 62.9 × 10^3^/*μ*L, making hyperleukocytosis/leukostasis an unlikely cause.

Both whole blood and plasma viscosity are elevated in patients with CML. Hyperviscosity syndrome and subsequent occlusion of blood vessels supplying the internal ear have been proposed in the pathogenesis of deafness in patients with CML [10]. Deafness secondary to leukemic infiltration of the cochlea has been reported in literature [11, 12]. Significant improvement was seen with intrathecal methotrexate administration in some patients [6, 11, 12]. However, conventional therapy and intratympanic steroid administration have not proved to be beneficial in these patients.

Thrombotic and hemorrhagic complications are seen in patients with myeloproliferative disorders. Factor V deficiency, anticardiolipin antibodies, and abnormal endothelial function have been associated with thrombotic complications in patients with CML and other myeloproliferative disorders [13, 14]. Beta-N-acetyl hexosaminidase is released from platelets during activation. Both content and release of beta-N-acetyl hexosaminidase are altered in patients with CML and thought to be associated with thrombotic/hemorrhagic complications [15]. Acquired vWF deficiency is associated with CML and other myeloproliferative disorders [16–19].

Platelet dysfunction is frequently seen in patients with CML and other myeloproliferative disorders [20–23]. Although defects in collagen, ristocetin, and arachidonic acid are seen in minority of patients, it is ADP aggregation that is mainly defective in these patients [23–25]. Defects in receptor function secondary to decrease in glycoprotein 1b and 2b/3a complex and subsequent low intracellular calcium influx have been associated with platelets dysfunction in patients with CML [26–28]. Abnormal Abelson kinase (abl) activity is also thought to play a role in platelet dysfunction [21]. In these patients resolution of platelet function defects was seen with imatinib therapy [21]. Lastly, hyperleukocytosis by itself can interfere with normal platelet function.

Therefore, both rheological and coagulation abnormalities can increase the risk of bleeding in patients with CML. Hemorrhagic complications such as cerebellar hemorrhage leading to hydrocephalus and subdural hematoma have been reported in patients with CML [29–31]. Our patient had excessive bleeding following bone marrow biopsy. Imaging of his brain postseizure and hearing loss showed multiple hemorrhages. It is likely that both rheological and coagulation abnormalities may have contributed to multiple hemorrhages in our patient's brain and subsequent deafness. CML as a rare cause of labyrinth apoplexy has also been reported in literature [32].

Recently, imatinib therapy has been associated with reduced levels of alpha 2-plasmin inhibitor and platelet dysfunction. Cases of subdural hematoma in patients treated with high dose imatinib therapy have been reported [30, 31]. Vocal fold hemorrhage in a patient with CML after imatinib treatment has also been reported [33]. Therefore, use of imatinib in patients with hemorrhagic complications secondary to CML is questionable.

Nilotinib is a second generation BCR-ABL tyrosine kinase inhibitor, used for the treatment of patients with chronic and accelerated phase Philadelphia chromosome-positive CML resistant or intolerant to treatment with imatinib. Side effects typically include rash, pruritis, headache, nausea, and vomiting. Grade 3/4 toxicities include thrombocytopenia, neutropenia, elevated lipase, hyperglycemia, and hypophosphatemia [34]. Cases of sensorineural deafness secondary to imatinib therapy have been reported in literature [35, 36]. Deafness as a side effect of nilotinib is not known. It is difficult to say whether nilotinib contributed to permanent deafness in our patient.

## 4. Conclusion

Deafness as an initial manifestation of CML is very rare. Cases have been reported with acute myelogenous leukemia and blast phase of CML. In these patients leukostasis secondary to hyperleukocytosis was the main cause. Our patient presented with sudden deafness when the white blood cell count had dropped to 62.9 × 10^3^/*μ*L, making hyperleukocytosis an unlikely cause. Imaging of his brain postseizure and hearing loss showed multiple hemorrhages. Therefore, it is likely that these hemorrhagic lesions may have caused permanent deafness in our patient. To our knowledge, this is the first reported case of permanent sensorineural deafness in a CML patient secondary to intracerebral hemorrhage.
